# Comparison of the capillary and venous blood plasma lipidomes: validation of self-collected blood for plasma lipidomics

**DOI:** 10.1016/j.jlr.2025.100755

**Published:** 2025-02-12

**Authors:** Ahsan Hameed, Mario G. Ferruzzi, Colin D. Kay, D. Keith Williams, Elaheh Rahbar, Andrew J. Morris

**Affiliations:** 1 Department of Pediatrics, University of Arkansas for Medical Sciences, Little Rock, Arkansas, USA; 2 Arkansas Children's Nutrition Center, Department of Pediatrics, University of Arkansas for Medical Sciences, Little Rock, Arkansas, USA; 3 Central Arkansas Veterans Affairs Healthcare System and University of Arkansas for Medical Sciences, Little Rock, Arkansas, USA; 4 Department of Biostatistics, University of Arkansas for Medical Sciences, Little Rock, Arkansas, USA; 5 Department of Biomedical Engineering, Wake Forest University School of Medicine, Winston Salem, NC, USA; 6 Department of Biomedical Engineering, Texas A&M University, College Station, TX, USA; 7 Department of Veterinary Physiology & Pharmacology, Texas A&M University, College Station, TX, USA; 8 Blood Biomarker Core of the CARE4Kids Research Consortium, Los Angeles, CA, USA

**Keywords:** capillary blood, Tasso+, Lipidomics, venous blood, microsampling, lipid biomarkers

## Abstract

Venipuncture of the upper extremities is commonly used to collect blood for plasma lipidomics. However, self-administered blood collection devices such as the Tasso+™ system for capillary blood sampling and plasma separation are convenient and enable frequent sampling without a clinical blood draw. The purpose of this study is to validate Tasso+ sampling for plasma lipidomics by comparing the venous blood and Tasso+-sampled capillary blood plasma lipidomes. Lipids are proven or putative biomarkers of human health and disease and indicators of nutritional and toxicological status. Because exchange of blood components including lipids occurs in capillaries, the capillary and venous blood lipidomes might be different, which could confound use of Tasso+-sampled blood as a surrogate for venous blood plasma. Here we compared the lipidomes of Tasso+-drawn capillary blood plasma and venous blood plasma in 10 male subjects using high-resolution mass spectrometry-based lipidomics. While there was substantial interindividual variability between lipidomes, comprehensive statistical approaches with cross-validation and multiple testing adjustments showed no difference (adjusted *P*-value > 0.05) in lipid composition of the paired blood samples. A linear regression model with Spearman correlation analysis also showed a significant-to-near-perfect level (r = 0.95–0.99) of concordance between the samples. Aside from monoacylglycerols and cardiolipins, every class of lipid was strongly correlated (r = 0.9–0.99) between paired venous and capillary blood plasma. In summary, the capillary and venous blood plasma lipidomes are essentially identical making self-administered collection of capillary blood a viable approach for clinical blood plasma lipidomics.

Lipids are a diverse group of biomolecules that serve as ubiquitous components in cellular membranes and also act as signaling mediators for various biological processes and play key roles in energy homeostasis in health and disease. Lipidomics, analysis of hundreds of lipid species in multiple classes after extraction from biological samples, is an emerging bioanalytical technique enabling the simultaneous identification and relative quantification of lipids (1). Cumulative evidence identifies certain circulating lipids as biomarkers of human health and status (2, 3). Lipid biomarkers have been correlated with dietary patterns to reveal the beneficial effects of specific nutritional regimens (4, 5, 6, 7, 8) and show promise for screening, early diagnosis, and therapeutic decision making in cardiometabolic diseases (9, 10), neurodegenerative disorders (11), and certain types of cancers (12). These studies use biofluids or tissues as a source of material for lipidomics.

Blood is a readily accessible and commonly analyzed biological fluid for preparation of plasma or serum used for lipidomics and for many other research purposes or laboratory tests. Collection of high-quality volumetric blood for these purposes is important as composition of blood provides a wealth of reliable information concerning a patient's health. Venipuncture is the clinical gold standard for blood collection. Phlebotomists use a needle to puncture a vein, usually in the arm. Patients can be uncomfortable with this procedure, and bruising is often a side effect (13, 14). Venous blood draws are not ideal for outpatient-based longitudinal clinical studies that require frequent repeated sample collection. Moreover, the recent global COVID-19 pandemic has forced both educational and healthcare institutions to reconsider this traditional blood collection approach because many subjects are hesitant to participate in clinical trials that require multiple blood draws for diagnostic testing. To address this, Tasso+ (Tasso+, Inc, Seattle, WA) recently introduced a patient-centric, single-use, clinical-grade, automatic at-home capillary blood collecting device called Tasso+ (formerly called Tasso+One™ hemolink) (https://www.Tassoinc.com) (15). Tasso+ is a novel collection system designed to collect up to 5 ml of blood from subcutaneous capillaries using gravity and light suction without the need for venipuncture. This capillary blood collection device simplifies the blood collection process to a single push of a button and uses open microfluidics to separate plasma from the collected whole blood (16). This device can be used by participants to collect stabilized blood plasma at home painlessly that can be shipped to laboratories for research and diagnostic purposes. A few studies have reported the use of Tasso+ in clinical and nonclinical settings (17, 18, 19), and the device has been assessed for clinical chemistry (20, 21, 22), hematology (23), and serology (24, 25), with good analytical performance and participant acceptance. However, there are no studies evaluating the suitability of Tasso+-based collected blood for lipidomics. Therefore, the aim of this pilot study was to establish and assess the suitability of Tasso+-drawn blood plasma as an alternative to venous blood plasma for clinical lipidomics.

## Materials and Methods

### Participants and study design

**Fig. 1:**
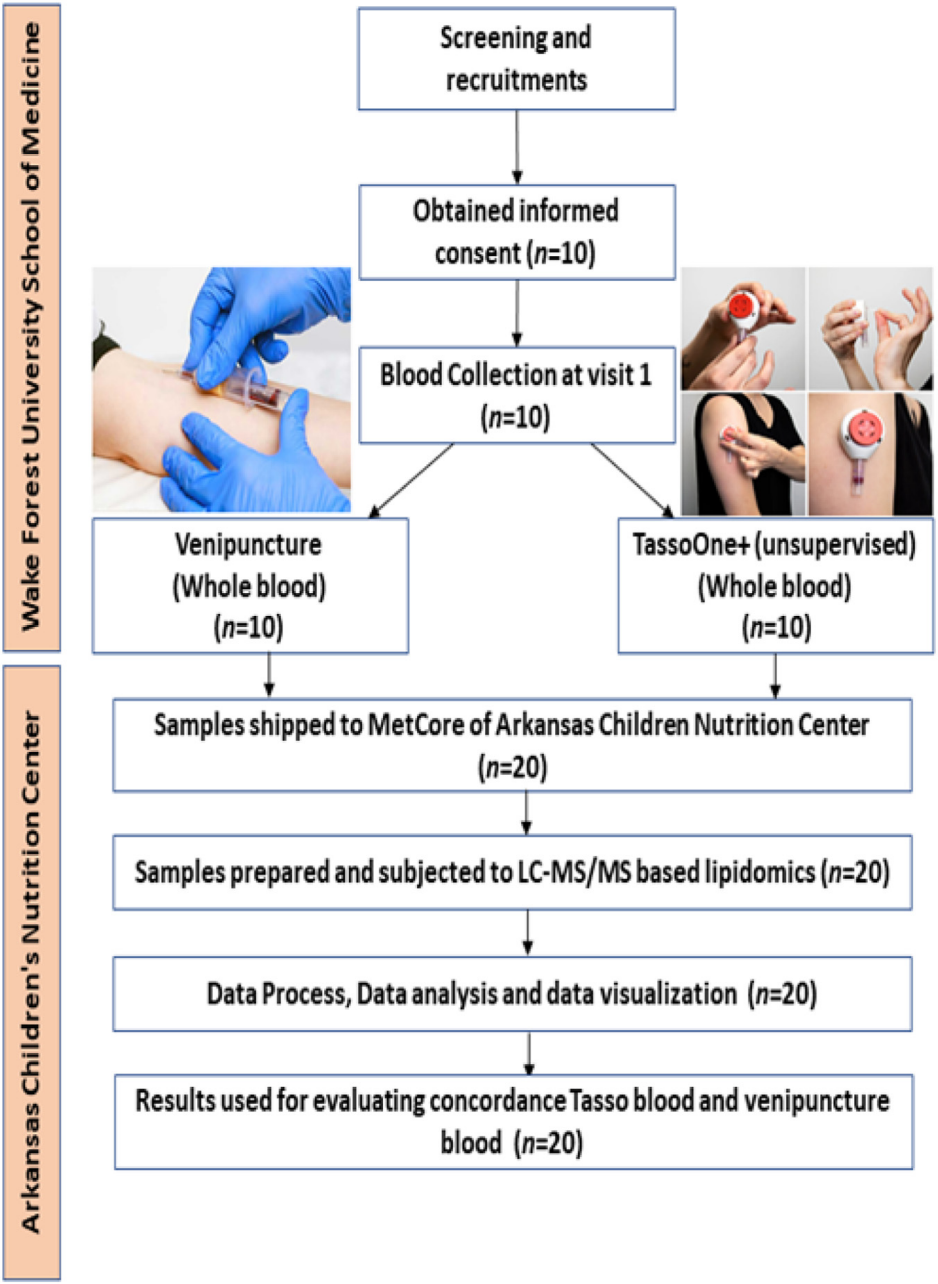
Study Design. Overall design of our study to investigate the lipid composition venipuncture blood plasma and capillary blood collected using the Tasso+ device.

Blood was collected from healthy (nonfasting) consenting adults (>18 years old) under an approved Institutional Review Board (IRB) protocol at Wake Forest University School of Medicine. The IRB protocol number for the healthy subject biospecimen collection at Wake Forest School of Medicine was IRB#00039804. The study abided by the World Medical Association Declaration of Helsinki Ethical Principles for Medical Research Involving Human Subjects. The study participant demographics and specimen collection data are outlined in Table 1. Subjects provided blood using traditional venipuncture and the TASSO+ device. In both methods, blood was drawn into an EDTA-containing tube. For venipuncture we used BD EDTA vacutainers (3 ml) and for the Tasso+ device we used a BD Microtainer® EDTA (light purple top) blood collection tube (0.25–0.5 ml) that was compatible with the TASSO+ device. Exclusion criteria were known pregnancy, any known inflammatory disease, a positive COVID-19 test or case within the past 2 weeks, and any aspirin or nonsteroidal anti-inflammatory drug use within the past 24–48 h. In this pilot we recruited ten healthy subjects (Fig. 1). Once collected, venous blood was inverted gently a few times to ensure mixing with EDTA and then was centrifuged at 1500 *g* for 15 min at 4°C to obtain plasma. Plasma was aliquoted and stored in cryovials at −80°C for future biomarker testing.

**Table 1:** Participant demographics

Category	Total
Total number of participants	
Gender-n	10
Male-n (%)	5 (50%)
Female-n (%)	5 (50%)
Age (years)-mean (range)	
Male (years)-mean (range)	30.8 (20–53)
Female (years)-mean (range)	29.1 (20–53)
Weight (kg)-mean (range)	
Male (kg)-mean (range)	>95
Female (kg)-mean (range)	>95
Race-n (%)	
White-n (%)	9 (90%)
Asian-n (%)	1 (10%)
Hispanic-n (%)	0 (0%)

### Blood collection

Each subject provided matched blood samples using both blood collection methods. A trained phlebotomist at Wake Forest University Medical Center performed traditional venipuncture to obtain venous blood samples. After venipuncture, blood was collected using the Tasso+ kit (Tasso+, Inc). The subject's arm was prewarmed using a disposable hand warmer (included in the Tasso+ kit) and then sanitized using an alcohol wipe. The Tasso+ device was removed from the sterilized packaging, the BD microtainer tube (EDTA) attached, and placed on the subject's upper arm. Once secured, the red button was pressed and capillary blood samples were obtained over the course of 2 min. Because the goal of the study was to establish concordance between venipuncture and capillary blood, all blood collections for this investigation were done at Wake Forest University Medical Center during a scheduled appointment at the same time for both venipuncture and Tasso+ blood collection.

### Sample preparation for lipidomics

Lipid extraction was performed in 4 ml borosilicate glass tubes with Teflon lined cap employing the modified Folch method. Briefly, plasma samples were thawed at room temperature and 50 μl extracted by addition to a mixture of 450 μl 0.1 M HCl, 2 ml MeOH, and 1 ml CHCl_3_ to give a single phase. The tubes were vortex mixed for 5 min, and then an additional 1 ml CHCl_3_ and 1.3 ml 0.1 M HCl were added to generate two phases. The tubes were vortex mixed again and then centrifuged at 3000 *g* in a swinging bucket centrifuge to separate the phases. The lower phase was removed to a 4 ml borosilicate glass vial using a Pasteur pipette and evaporated to dryness under nitrogen gas using a BioTage LV Turbovap solvent evaporator. At the beginning of the extraction, each sample was spiked with 10 μl lipid internal standard purchased from Avanti (Splash Lipidomix, Alabaster), which contains phosphatidylcholine 15:0–18:1(d7) PC, phosphatidylethanolamine 15:0–18:1(d7) PE, phosphatidylserine 15:0–18:1(d7) PS, phosphatidylglycerol 15:0–18:1(d7) PG, phosphatidylinositol 15:0–18:1(d7) PI, phosphatidic acid 15:0–18:1(d7) PA, lysophosphatidylcholine 18:1(d7) LPC, lysophosphatidylethanolamine 18:1(d7) LPE, cholesteryl ester 18:1(d7) CE, monoacylglycerol 18:1(d7) MG, diacylglycerol 15:0–18:1(d7) DG, triacylglycerol 15:0–18:1(d7)-15:0 TG, sphingomyelin 18:1(d9) SM, and Cholesterol (d7). Dried samples were reconstituted in MeOH stored at −80°C until analysis. Two quality control (QC) samples were prepared by separately pooling the venous or Tasso+ blood samples from every subject.

### Untargeted lipidomic analysis

A Dionex UltiMate 3,000 ultra high-performance liquid chromatography (UHPLC) system (Santa Clara, CA) coupled to an electrospray ionization source (ESI) with a Q Exactive™ MS instrument (Thermo Fisher Scientific) was used for the untargeted lipidomic analysis. For data acquisition, 10 μl aliquots of sample solution, maintained at 4°C in an autosampler, were injected onto a reversed phase Waters ACQUITY UPLC BEH C8 (50 mm × 2.1 mm, 1.7 μm) maintained at 40°C by gradient elution. Mobile phase A was 40% acetonitrile in water and mobile phase B was isopropanol:acetonitrile (9:1), both containing 10 mM ammonium formate and 0.1% formic acid. The flow rate was 0.250 ml.min^−1^, with the elution gradient as follows: 0–24.00 min, 32% B; 25.0–28.0 min, 32%–97% B; 29.0–29.1 min, 97%–32% B; 29.2–35.0 min, 32%–32% B. Separate datasets were acquired in positive and negative ionization modes using a heated electrospray ionization source. The source and ion transfer parameters applied were as follows: spray voltage 3.5 kV (positive) and 2.8 kV (negative). For both ionization modes, the sheath gas, aux gas, capillary temperature, and heater temperature were maintained at 12, 5 (arbitrary units), 320°C and 300°C, respectively. The S-Lens RF level was set at 60. The Orbitrap mass analyzer was operated at a resolving power of 70,000 full width at half maximum (*m/z* 200) in full-scan mode (scan range, 80–1,200 *m/z*; automatic gain control target, 5 × 10^6^) and 15,000 in the Top 5 data-dependent MS2 mode (stepped normalized collision energy, 20; injection time, 250 ms; isolation window, 1.0 *m/z*; automatic gain control target, 5 × 10^6^) with a dynamic exclusion setting of 6.0 s. LC-MS grade methanol was used to minimize intensity of the background signal. Product ion scans in positive and negative ion modes were performed on each sample, to maximize numbers of lipid species identified. Sampling order was randomized prior to analysis.

### Quality control

Quality control measures included randomization of the samples within the acquisition sequence; injection of QC pool samples at the beginning, end, and between every eight actual samples for a specific matrix; analysis of method blanks; and analysis and filtration of data based on the data of pooled QC sample and control for variability of chromatographic peak shapes, retention times, and intensities of the internal standards.

### Data processing

The LC-MS instrumental files generated during lipidomic analyses were processed using MS-DIAL v. 4.9.221218 software with the following parameters: (i) data collection: MS1 tolerance, 0.01; MS2 tolerance, 0.025; (ii) peak detection: minimum peak height, 15,000; mass slice width, 0.05; smoothing method, linear weighted moving average; smoothing level, 2; (iii) MS/MS identification setting: accurate mass tolerance (MS1), 0.005; accurate mass tolerance (MS2), 0.005; identification score cutoff, 80%; (iv) alignment: retention time tolerance, 0.05 min; MS1 tolerance, 0.01 Da; peak count filter, 5%; gap filling by compulsion, true, Adduct ions: +H and +NH_4_ for positive ion mode, and −H, -HCOO and −2H for negative ion mode. Lipids were annotated based on retention time–*m*/*z* match from MS-DIAL internal MSP library that contains the theoretical MS/MS spectra of lipids (LipidsMsmsBinaryDB-VS68-FiehnO by Oliver Fiehn laboratory). Some complex lipids were annotated using in silico MS/MS spectra available in MS-DIAL software. Exported data sets for each matrix and platform as signal intensity from the detector (peak areas) were further filtered by removing lipids with (i) a max sample peak height/blank peak height average < 10, (ii) an *R*^2^ < 0.8 from a dilution series of QC sample, and (iii) a relative standard deviation (RSD) > 20% from QC samples injected between 10 actual study samples. Data from the ionization mode with higher annotation rates for lipids that ionized in both modes were used.

### Lipid identification and reporting

The lipidomics dataset was filtered to select only those lipid features detected in >80% of QC samples with RSD < 30%. Following the QA and QC filtrations, only those lipids were used for statistical analysis whose identity was confirmed using the MS/MS spectra and all other lipid features without matching MS/MS were removed.

### Multivariate analysis

Multivariate analysis was performed using MetaboAnalyst 5.0 (http://www.metaboanalyst.ca). Statistical models were created for the TIC normalized data (median) after logarithmic transformation (base 10) and Pareto scaling. AP [principal component analysis (PCA)] modeling was first used to evaluate the ability to predict the different groups. Next, we relied on this statistical analysis to establish an appropriate classification model by using the data obtained from samples and to identify key factors that lead to the sample differences. The partial least squares-discriminate analysis (PLS-DA) explored the differences between features from different groups by incorporating the known classification and indicated the variable importance of features with a loading plot (indicating the responsible variable for deviations from normal operation) according to variable importance plot (VIP). The statistically significant features responsible for discrimination of three groups were obtained from VIP (VIP > 1.0) in the projection Score from the PLS-DA model and *P*-value (significance at *P* ≤ 0.05) based on volcano plot. The volcano plot shows the significance and the fold change of each identified lipid. The significant lipids were selected by the volcano plot with a fold change threshold > 1.5 (or < 0.5) and *P*-value < 0.05 using the Mann-Whitney test with the Benjamini-Hochberg false discovery rate (FDR) multiple testing correction. The cross-validation and permutation tests were used to evaluate the robustness of the model.

### Univariate analysis

Univariate analysis was performed to compare lipidomic data sets from matched venous and capillary blood with MetaboAnalyst 5.0 using an unpaired two-sided *t* test on the log-transformed and median-based normalized data. Significant features were obtained by cutoffs of raw *P*-value at 0.05, adjusted *P*-values, and FDR at 0.05. Robustly differential features considered were defined by a fold-change (FC) threshold of 1.5 and adjusted *P*-values ≤0.05.

### Correlative and regression analysis, hierarchal clustering

We computed the Pearson's correlation coefficient and hierarchical clustering analysis (HCA) to evaluate the correlation between matched capillary and venous blood lipidomes. Both Pearson and HCA analysis were carried out by MetaboAnalyst 5.0. Additionally, we employed basic linear regression to derive the regression line between matched capillary blood (dependent variable) and venous blood lipidome datasets (independent variable) using Jamovi statistical software (Version 2.5.6) and the simple slop test of interactions was automatically generated. The fraction of the capillary blood lipidome that could be explained by the venous blood lipid data was also examined using the *R*^2^ statistic. We estimated an almost perfect level of agreement (0.99), significant (0.95–0.99), moderate (0.90–0.95), and low (<0.90) agreement using the concordance correlation coefficient of Lin (rc) to assess the degree of agreement between the two methods (26). We also used Jaccard similarity index (which ignores possible biasness and considers overlap in lipids detected) to compute the similarity between the lipid measurements in matched venous and capillary bloods, considering an almost perfect level of agreement (0.99), substantial (0.95–0.99), moderate (0.90–0.95), and poor (<0.90) agreement. Jaccard similarity index, symbolized by *J*, is calculated using the formula (27):

$$Jaccard\;Index=\frac{\left(the\;numbers\;in\;both\;sets\right)}{\left(the\;numbers\;in\;either\;set\right)}*100$$

To gain further insight into the systematic differences in measurements between the two groups, the Bland and Altman mean difference with upper and lower 95% confidence interval (CI) was calculated and used as an estimate of the absolute bias. The relative bias discussed here refers to the comparison between the response values of the two groups (Tasso+ and the venipuncture group) across different classes. Specifically, this bias is calculated as the percentage difference between the two groups for each class, using the formula:

$$\text{Relative}\;\text{bias}\;\left(\%\right)=\frac{\text{Mean}\;of\;\left(tasso-Other\right)}{Mean\;of\;\left(tasso+other\right)/2}*100$$

This statistic directly compares the average response values of the two methods, showing whether one method systematically produces higher or lower readings than the other.

## Results

### Lipid composition of capillary and venous blood plasma

Comprehensive lipidomics analysis of blood plasma samples collected by venipuncture or the Tasso+ device was done as described in the methods. Plasma samples were analyzed in both ionization modes resulting in two datasets having total 25,465 and 19,148 identified features (each from ESI^+^ and ESI^-^). Both datasets were subjected to data QA and QC procedure selecting features detected in >80% of QC samples with RSD <30%. Following the QA and QC filtrations, lipids were identified from the remaining features using spectral database searching and features without matching MS/MS were removed. The remaining two datasets contained 824 and 687 lipid features with level II identifications, which were used for downstream statistical analysis. The predominant lipids identified were from 14 lipid classes, namely, phosphatidylcholine (PCs), lyso-PC (LPC), phosphatidylethanolamine (PEs), lyso-PE (LPE), phosphatidylinositol (PIs), lyso-phosphatidylinositol (LPI), monoacyl/alkylglyceride (monoglyceride) (MG), diacyl/alkylglyceride (diglyceride) (DG), sphingomyelin (SM), sterols (ST), triacyl/alkylglycerides (triglycerides) (TG), phosphatidylglycerol (PG), acylcarnitines (AC), phosphatidylserine (PS), and sterol esters (SE). Details are provided in supplemental Spreadsheet S1. We also analyzed pooled Tasso+-collected capillary blood plasma and venous blood plasma samples from the entire study cohort. We used the mass labeled lipid class specific internal standards for quantification of all lipid species detected in classes where a standard was included. The data are presented in supplemental Table S1. Lipid class-specific concentrations are broadly comparable with those reported by others (28, 29). There were no significant differences in pooled venous and Tasso+ plasma levels of the lipid classes that we quantified.

**Fig. 2:**
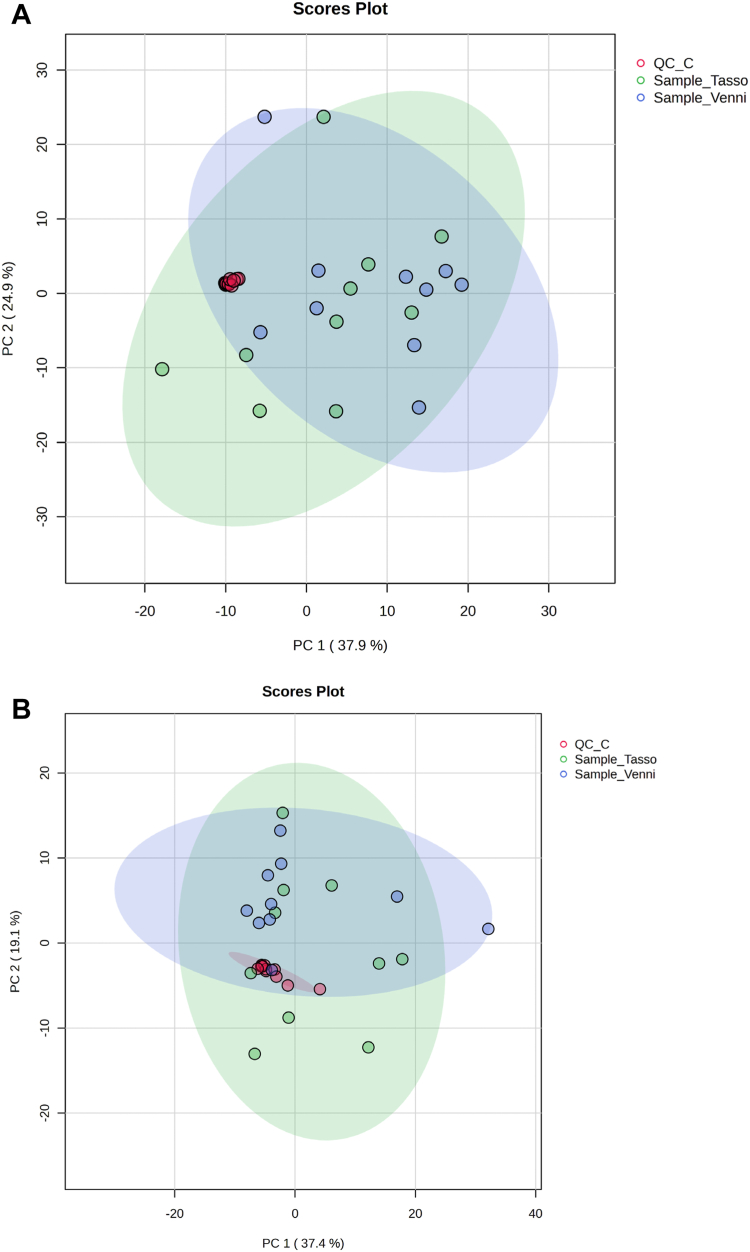
Principal Component Analysis. Scores plot of PCA from positive (A) and negative modes (B), showing overlap between matched venous and capillary blood samples from healthy adults, indicating near-complete overlap (n = 20, for both). Scores plot of PCA of the plasma metabolome from venipuncture-collected blood (blue, n = 10) and Tasso+-collected blood (green, n = 10). Shaded circles represent 95% CIs, while colored dots illustrate individual samples. The axes are labeled by the first and second principal component (PC 1 and 2, respectively) with the percentages of variance of the data explained by that principal component in parentheses. Statistical models were created for the TIC normalized data (median) after logarithmic transformation (base 10) and Pareto scaling.

The identified lipid features were normalized to the median of total signals of samples, log transformed (base 10), and Pareto scaled before using for unsupervised analysis. The unsupervised analysis revealed the broader clustering of samples from both matched venous and capillary blood samples from healthy adults based on the plasma lipidome (Fig. 2). The broader clustering represents the range of classes of lipids present in samples, whereas the significant overlap represents the high level of concordance between capillary and venous blood lipidomes. The total variance of the first two principal components contributed 62.8% and 49.2% for both polarities in the PCA model for matched venous and capillary blood samples groups (ESI+, PC 1 = 37.9%, PC 2 = 24.9%; ESI-, PC 1 = 36.5%, PC 2 = 12.7%). The unsupervised multivariate analysis revealed no significant differences or discernible pattern between the 2 groups by sample type.

To further search for features that might discriminate between capillary and venous blood, a partial least squares discriminant analysis (PLS-DA) as a subsequent supervised multivariate data analysis method was built (Fig. 2). In these PLS-DA models, the first two components contributed to a total variance of 22.5% and 47.4% for the data acquired in both positive and negative modes (ESI+, PC 1 = 16.3%, PC 2 = 6.2%; ESI-, PC 1 = 36.3%, PC 2 = 11.1%), respectively. The PLS-DA models could not separate the matched venous and capillary blood groups based on the lipid datasets. The model was assessed by monitoring the diagnostic statistical like model goodness of fit (R2) and predictive ability (Q2) values. The model validation of supervised multivariate data analysis showed satisfactory goodness of fit of model for both modes (positive, R2 = 0.425, negative, R2 = 0.38), but predictive ability of model was negligible and/or overfitted (positive Q2 = -0.461, negative, Q2 = -0.295). The negative Q2 values indicated the nondiscriminative power of models suggesting the all samples belonged to the same class of controls as elaborated in the work of Szymańska, Saccenti, Smilde, Westerhuis (30). Furthermore, since the values of the diagnostic statistics can be assumed to correspond to good models with high discriminative power, the values of these diagnostic statistics can be obtained completely at random (31), which shows inability to know which values of these diagnostic statistics actually correspond to good discrimination between capillary and venous blood samples. To overcome this issue, a 1,000 permutation test was performed, which is described as a tool to measure the level of uncertainty of the diagnostic statistics (32). The results of the 1,000 permutation test on this dataset showed that the model produced by PLS-DA was valid with respect to model goodness of fit (*P* = 0.01); however, predictive ability (Q2) of the model was invalid (*P* = 0.476) indicating lower and/or no discriminatory power of model, or no significant feature based on this model was seen. Biologically, this makes sense as we expected minimal and/or insignificant differences between the lipidomes of matched venous and capillary blood samples. Furthermore, it is also possible from a statistical analysis perspective to observe something (in)significant in multivariate analyses that is not (in)significant in univariate tests or vice versa due to several reasons stated by Saccenti, Hoefsloot, Smilde, Westerhuis, Hendriks (33). We also carried out conventional univariate data analysis (*t* test and nonparametric tests) on this dataset, which also produced no significant results. Specifically, these data were analyzed in MetaboAnalyst 5.0, and *t* test and nonparametric tests identified zero significant features, even at an FDR cutoff of 0.1. Using every available method of dealing with missing values in MetaboAnalyst 5.0 (there are 9 in total: exclude features with missing Metabolites 2019, 9, 90 9 of 19 values that were replaced by a mean/median/minimum, or were imputed using tools provided by MetaboAnalyst 5.0) led to models that produced no significant results in the lipidomes comparison of matched venous and capillary blood samples.

### Concordance, hierarchal clustering, and regression analyses

**Fig. 3:**
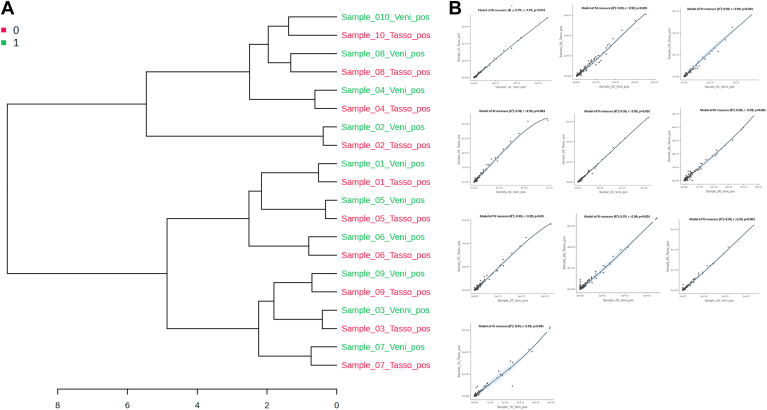
Correlation analysis of venous and Capillary Blood lipidomes. A: HCA dendrogram showing the relationship between the complete lipidomics datasets matched participant's blood samples using Pearson distance and Ward clustering. B: Correlation between paired capillary and venous blood sample collections from the donors (n = 10) followed by the generation of “lines of the best fit” and corresponding R2 values for each matched capillary and venous blood sample. The axes are relative abundances (integrated peak areas, arbitrary units). Spearman correlations were also performed on matched lipidomics dataset of each participant. Spearman correlations coefficients are denoted by “r,” and probability values are represented by *P*-values. The Spearman correlations coefficients (r) for all matched samples were >0.95 with *P*-values <0.001 showing perfect-to-significant level of concordance between sample pairs.

Given that our results suggest high degrees of similarity between the lipidomics datasets of match venous and capillary drawn blood samples, we next pursued several correlative statistical approaches to find and establish the level of concordance between the plasma lipidomics datasets of matched venous and capillary blood plasma. A hierarchical cluster analysis (HCA) dendrogram was created from the entire lipidomics dataset using the Ward clustering methods and Pearson distance measurements to visualize the differences between the various samples as well as the similarities between the (paired) samples. As can be seen in Fig. 3A, the HCA dendrogram clearly clustered the matched samples together, demonstrating the significant correlations between the paired samples. The paired sample clustering indicates a strong degree of similarity in the lipidomics datasets of the subjects' matched blood samples. This shows the high concordance between the venous and blood plasma lipidomes persists despite the substantial individual variation in plasma lipidomes of our study subjects, which is in turn consistent with observations reported by others (34). Linear regression analysis was carried out, and the linear scatterplots of each paired venipuncture versus Tasso+ -collected capillary blood (n = 10) with the slope, model fit measures (*R*^2^), Pearson's correlation coefficient (r), and 95% CI are shown in Fig. 3B. The dots represent the individual lipids, solid black line depicts the linear regression of the actual data, and light blue shade around the black solid lines shows the standard error for the collected data. Linear scatter plots show perfect agreement between the paired blood samples as slope of all linear fit was ≥0.95 with the 95% CI *P* = <0.001. The linear regression analysis of 7 paired samples showed perfect regression coefficient r = ≥0.99, whereas the rest of three paired samples showed significant correlation (r = 0.95–0.98). The variation in the regression coefficient represents the interindividual variations in the lipidomes. This comparatively low variations show that interindividual variations are higher than the lipidomics variations between capillary and venous bloods. Similarly, the Pearson's correlations demonstrated near perfect correlation (r value ≥ 0.99, *P* < 0.001) between the paired samples signifying the perfect concordance between them (Fig. 3B).

## Discussion.

Several published studies have investigated use of Tasso+-drawn capillary blood for high-throughput metabolomics (34, 35), proteomics (36, 37), transcriptomics (38), and polyfluoroalkyl-omics (39). Saito, Ueno, Nakayama, Nitta, Arai, Hasunuma, Saito (35) demonstrated a larger impact of interindividual variation compared with the lipidomics differences between capillary and venous blood as presented herein. While this study provides evidence of the highest level of similarity (*R*^2^ = 0.99) in the lipid levels between capillary and venous blood plasma, 57 (11.4%) lipids were significantly different and most of these belonged to the PE and DG classes. In another quantitative metabolomics study, Catala, Culp-Hill, Nemkov, D'Alessandro (34) measured the level of amino acids, energy-related metabolites (glycolytic and Krebs cycle intermediates, redox-related metabolites), acyl-carnitines and bile acids in paired capillary and venous blood draws and reported an overall median correlation coefficients>0.7 for all the tested metabolites. These authors found a substantial level of similarity between the blood draws for bile acids and acyl-carnitines but reported comparable measurements for metabolites related to redox homeostasis (e.g., cysteine and glutathione), which the authors attributed to an overall decreased level of oxidation for capillary blood. In one proteomics study, the agreement between conventional venipuncture and Tasso+ SST (prior generation Tasso+ device) with immediate on-site processing for 17 protein inflammatory biomarkers was evaluated. The majority of the proteins, including PCT, IL-6, D-dimer, IL-1B, and IL-1Ra, showed good correlations with little sample type bias (37). Another large-scale targeted proteomics study measuring 384 proteins and including an inflammatory panel (e.g., cytokines and chemokines) in paired blood draws reported higher degree of interindividual protein variance and thus weak correlations (36). This work observed only a small fraction of proteins (14.8%) showing stronger correlations (Pearson r ≥ 0.5) with low variance (coefficient of variation ≤ 0.20) after 24 h of blood collection. The authors further proposed that low correlations for the remaining proteins may be caused by delayed blood sample processing, which can result in cellular lysis, microthrombosis, and protein degradation and that an on-site blood processing after Tasso+ blood collection might have improved the protein correlations. Capillary blood has also been validated for whole blood transcriptomics and compared with the venous blood transcriptome revealing high gene expression level concordance between the two sample types (38). Similarly, the levels of per- and polyfluoroalkyl substances (PFASs) have been determined in the self-collecting capillary blood and compared with the levels in venous blood (39). Regression models showed that concentrations in capillary blood were highly correlated with levels in venous blood and can be used to predict PFAS concentrations in venous blood. For most PFASs, these models produced slope factors that were extremely close to one (0.98−1.19 for venous vs. capillary whole blood measurements).

### Correlation of all lipid classes between venous and capillary blood

Because lipids from different lipid classes have traditionally been designated as biomarkers for the diagnosis of pathogenesis, progression, and severity of lifestyle diseases (40, 41), we sought to report the degree of similarity between each lipid class of paired lipidomes after establishing the concordance between the individual lipidome datasets of matched participants. Furthermore, there have been reports of possible capillary blood contamination with interstitial and intracellular fluids (42) that might result in differences in lipid content. Variability between capillary and venous blood plasma concentrations of glucose, pyruvate, succinate, reduced glutathione, cysteine, albumin, urea/urate, RBC counts, and hemoglobin has also been reported (34, 43, 44, 45). Previous research has demonstrated the validity of utilizing capillary blood for standard lipid panel measures (e.g., TG and lipoproteins) (46, 47). However, currently there are no conclusive results in the literature regarding the use of capillary blood sampling as a trustworthy substitute technique for all types of lipid measures. This work is the first to show that venous and capillary blood can be used interchangeably for plasma lipidomics. The correlations of all lipid classes with three distance indices (i.e., Spearman, Jaccard, and Lin) and possible relative bias between venous and capillary blood are shown in Table 2. Overall, the Lin concordance test showed stronger class-wise correlation compared with Spearman correlation and Jaccard similarity index.

**Table 2:** Correlation coefficients and concordance correlations coefficients of matching levels of capillary blood collected via Tasso+ device and venous blood from venipuncture in matched healthy individuals

Class	Spearman correlation	Jaccard Similarity index	Lin concordance Correlation (L_c_)	Relative Bias (%)	Bias CI (Lower)	Bias CI (Upper)	Bias *P*-values	Spearman *P*-values
PC/LPC	0.92	0.85	0.97	5.89	0.20	11.99	0.52	<0.001
PE/LPE	0.89	0.79	0.90	−9.53	−20.20	1.16	0.51	<0.001
PI/LPI	0.82	0.67	0.99	−1.02	−9.52	7.68	0.80	<0.001
TG	0.95	0.87	0.98	−1.11	−6.89	4.83	0.71	<0.001
DG	0.81	0.76	0.87	−7.30	−16.82	2.43	0.53	<0.001
Cer	0.84	0.82	0.95	−4.61	−17.25	8.59	0.58	<0.001
SM	0.98	0.90	0.99	4.01	1.72	6.44	0.49	<0.001
MG	0.50	0.48	0.45	−1.83	−18.68	15.05	0.80	<0.001
Car	0.90	0.90	0.88	1.77	−5.56	9.14	0.67	<0.001
CE	0.79	0.67	0.95	−31.78	−56.11	−7.15	0.51	<0.001
ST	0.72	0.59	0.90	−33.38	−51.79	−15.60	0.51	<0.001
CL	0.72	0.64	0.76	−12.86	−44.48	15.91	0.56	<0.001
FA	0.85	0.74	0.91	2.53	−2.89	8.56	0.55	<0.001

Phosphatidylcholines, both PC and LPC, were very similar between the capillary and venous blood draws with a moderate-to-substantial level of correlative agreement (0.9–0.99) that was statistically insignificant (*P* = 0.52) relative bias of 5.89%. TG and SM also showed substantial correlative scores (0.95–0.99) with statistically nonsignificant and negligible relative bias (Table 2). Previously, Catala, Culp-Hill, Nemkov, D'Alessandro (34) observed the identical level of strong averaging median correlations of 0.94 for palmitate, a TG, and sphingosine 1-phosphate, between venous and capillary blood in one quantitative metabolomics investigation with a bile acid panel. The Bland and Altman mean difference showed a relative bias of −7.30% for DG, demonstrating venous blood tends to have 7.30% higher levels of DG than capillary blood, but this difference was statistically nonsignificant (*P* = 0.53). (Acyl)Carnitines (Car), mitochondrial biomarkers of genetic inborn errors of metabolism, exhibited sufficient correlations (0.9–0.95) in Spearman and Jaccard similarity index but Lin concordance correlation showed weaker similarity between venous and capillary blood samples. The relative bias for the (acyl)carnitines was statistically insignificant (*P* = 0.67) demonstrating that difference in (acyl)carnitines between the blood draws is likely due to random variations. Similar results published by Catala, Culp-Hill, Nemkov, D'Alessandro (34) reported that the acylcarnitine composition of venous and capillary blood plasma was comparable, with overall median correlation coefficients>0.95. Lin concordance test showed almost similar levels of both PI/LPI and PE/LPE in capillary and venous blood, as a strong correlative relationship (L_C_ = 0.9–0.95) was observed; however, two other indices showed lower correlations (<0.90). Despite being statistically insignificant, the relative bias for PE/LPE was around −9.53% indicating Tasso+-collected blood tends to contain 9.53% lower PE/LPE compared with the venous blood. In an untargeted metabolomics study, Saito, Ueno, Nakayama, Nitta, Arai, Hasunuma, Saito (35) also reported that levels of PE like PE(38:5e), PE(40:5e), [PE(18:1e/22:4)], and PE(40:5e), [PE(20:1e/20:4)] were higher in venous blood samples. On the other hand, PI/LPI tends to show much less relative bias, i.e., −1.02%, indicating the Tasso+ group provides a 1.02% lower reading then venous blood for LP/LPI. Ceramide levels between venous and capillary blood were strongly correlated (L_C_ = 0.95) and showed good agreement with insignificant level of relative bias (−4.61%). Sterols and cholesterols esters (CE) were the only two lipid classes that showed stronger correlative agreement between the venous and capillary blood but with relatively higher relative bias (ie, −33.38% and 31.78%). These differences were not statistically significant, which likely reflects the small number of sterols measured. Plasma levels of FA were also similar (L_C_ = 0.91) between the blood draws with statistically insignificant bias. The insignificant relative bias of 2.53% shows that Tasso+-drawn blood may contain 2.53% higher FA than FAs in the venous blood. Similar results were reported by Saito, Ueno, Nakayama, Nitta, Arai, Hasunuma, Saito (35), which found FA(18:1) and FA(18:2) were higher in capillary blood samples. In summary, there is moderate-to-significant concordance for most lipid classes observed between venous and capillary bloods. The reasons for the modest differences in some lipid classes, such as CL, MG, and DG, whose correlative agreement was lower than 0.9, are unknown, but they may be related to inherent lipid exchange in capillaries and variations in the relative abundance of these lipids in capillary blood relative to venous blood, or to bias in sample collection. Furthermore, because of the smaller sample size in this stratified study, we are cautious about interpreting the slight differences observed.

The present study has several strengths. As far as we are aware, this is the first study to explicitly assess lipids by comparing plasma obtained with the Tasso+ device with venous plasma. Our findings suggest that Tasso+-collected blood plasma is a suitable surrogate for venous blood plasma lipidomics. Because the capillary blood samples collected with Tasso+ and venous blood in our study were centrifuged and processed in the lab in less than four hours, there was less chance that outside factors like processing time or temperature fluctuations would affect lipid levels or composition of the samples. Future research should continue to assess the stability and dependability of lipids measurement utilizing the Tasso+ and other self-collection tools. The primary weaknesses of our study are the relatively small sample size and nonfasting state of our subjects. Replication with a larger study group would be helpful, although the correlations observed with only ten subjects was extremely high. Other concerns are that we used acidified organic solvents for extraction of plasma lipids, which, while offering optimal efficiency, is known to result in de- or transesterification and acid hydrolysis of some lipids (48). There might also be differences in lipid species that our mass spectrometry methods cannot detect or resolve. Follow-up research is needed to broaden the lipidomics coverage to include lipid (sub)classes other than those examined in the present work and to confirm the results using more quantitative methods, for example, targeted measurements of lipids with a broader range of internal standards.

## Conclusions

Capillary blood plasma samples collected with the Tasso+ device contained comparable levels of endogenous lipids with insignificant bias to matched venous plasma samples collected using venipuncture in nonfasting individuals. These findings demonstrate that the Tasso+ device is a reliable alternative method of blood plasma collection for nutritional lipidomics and potentially other lipid biomarker studies. Adopting this approach would simplify the monitoring of lipid biomarkers of diseases and dietary regimens and allow the surveillance of at-risk populations, particularly those in remote and rural settings. These findings pave the way for exploring the use of this device for more widespread lipid measurements, particularly when access to appropriate testing is limited by disability, distance, travel barriers, lack of trained phlebotomists, safety concerns, or other factors.

## Data availability

The lipidomics data described in the article, its supporting information, raw MS, and MS/MS data have been deposited to the Metabolomics Workbench where it has been assigned Study ID ST003576. The data can be accessed directly via its Project digital object identifier (DOI) of “https://doi.org/10.21228/M8GG1F”.

## Supplemental data

This article contains supplemental data.

## Conflict of interest

Tasso+, Inc. did not provide any funding or support for this work. The authors declare that they have no conflicts of interest with the contents of this article.
