# Supplementary material for: Comparison of the capillary and venous blood plasma lipidomes: validation of self-collected blood for plasma lipidomics
Source: J Lipid Res. 2025 Feb 12;66(3):100755. doi: 10.1016/j.jlr.2025.100755 (PMC11932689; doi:10.1016/j.jlr.2025.100755)
Supplement: Table S1 [file mmc2.docx]

| Lipid Class | Pooled Venous Plasma | | Pooled Capillary Plasma | |
| --- | --- | --- | --- | --- |
|  | Sum (nmol/ml) | | Sum (nmol/ml) | |
|  | Mean | SD | Mean | SD |
| TG | 2130.32 | 248.72 | 1896.44 | 211.01 |
| DG | 57.10 | 3.34 | 52.09 | 4.21 |
| MG | 9.01 | 0.55 | 8.31 | 0.94 |
| PE | 570.66 | 66.01 | 543.66 | 53.52 |
| LPE | 44.40 | 4.15 | 43.23 | 4.41 |
| PC | 1998.56 | 121.21 | 2075.34 | 150.79 |
| LPC | 149.11 | 14.51 | 165.89 | 18.02 |
| PS | 8.25 | 0.69 | 9.71 | 1.06 |
| PG | 8.20 | 0.69 | 7.87 | 0.84 |
| PI | 37.22 | 3.57 | 37.36 | 3.54 |
| SM | 420.09 | 23.34 | 458.65 | 54.85 |

**Table S1 Quantification of the indicated classes of lipids in pooled venous and plasma samples using lipid class specific internal standards.** Data are means +/- SD for four separate data acquisitions with each pooled sample. There were no significant differences (P>0.05) in summed lipid class specific lipid concentrations between the pooled venous blood plasma and capillary blood plasma lipid concentrations when compared using an unpaired t-test.
